# The *BIM* deletion polymorphism is a prognostic biomarker of EGFR-TKIs response in NSCLC: A systematic review and meta-analysis

**DOI:** 10.18632/oncotarget.4678

**Published:** 2015-07-27

**Authors:** Wei Nie, Xia Tao, Hua Wei, Wan-sheng Chen, Bing Li

**Affiliations:** ^1^ Department of Respiratory Medicine, Shanghai Changzheng Hospital, Second Military Medical University, Shanghai 200003, China; ^2^ Department of Pharmacy, Changzheng Hospital, Second Military Medical University, Shanghai 200003, China

**Keywords:** Bcl-2-like protein 11, non-small cell lung cancer, epidermal growth factor receptor, tyrosine kinase inhibitor

## Abstract

The prognostic value of Bcl-2-like protein 11 (BIM) deletion polymorphism for epidermal growth factor receptor (EGFR) tyrosine kinase inhibitors (TKIs) treatment in non-small cell lung cancer (NSCLC) were reported. However, the results remained controversial. Thus, we did this systematic review and meta-analysis to address this issue. Databases including PubMed, Embase, and the Cochrane Register of Controlled Trials were searched to find relevant studies. The primary outcome was progression-free survival (PFS). Five retrospective cohort studies were included. All of the studies were conducted in Asian population (n = 951). The methodological quality of all included studies was high. Compared with *BIM* wild type, *BIM* deletion polymorphism was predictive of shorter PFS in NSCLC patients who were treated with EGFR-TKIs (adjusted HR = 2.38, 95% CI 1.66–2.41, *P* < 0.001). In conclusion, the *BIM* deletion polymorphism was associated with poor response in NSCLC patients who received EGFR-TKIs treatment.

## INTRODUCTION

Epidermal growth factor receptor (EGFR) tyrosine kinase inhibitors (TKIs) became the first-line treatment for patients with non-small cell lung cancer (NSCLC) harboring mutant *EGFR* gene [1]. Most of these patients are highly responsive to treatment with EGFR-TKIs. However, about 20–30% patients with EGFR activating mutations show primary resistance to EGFR-TKIs [2]. Therefore, identifying the prognostic biomarkers of primary resistance to EGFR-TKIs in these patients is urgently needed.

Bcl-2-like protein 11 (BIM) is a BH3-only proapoptotic member of the Bcl-2 protein family [3]. Up-regulation of BIM correlated with gefitinib-induced apoptosis in gefitinib-sensitive *EGFR*-mutant lung cancer cells [4]. In addition, knockdown of BIM expression by RNA interference eliminated erlotinib-induced cell killing *in vitro* [5]. Addition of a BH3 mimetic significantly enhanced killing of NSCLC cells by gefitinib [6]. Furthermore, high level of BIM expression was a marker of longer progression-free survival (PFS) in *EGFR*-mutant NSCLC treated with erlotinib [7]. Therefore, BIM might be a biomarker of survival in *EGFR*-mutant NSCLC.

Recently, King Pan Ng and colleagues identified a common intronic deletion polymorphism in *BIM* [8]. This *BIM* deletion polymorphism was absent in individuals from African and European populations, but was found in about 12% of Asian population [8]. They demonstrated that patients with *EGFR*-mutant NSCLC harboring *BIM* deletion polymorphism showed significant inferior responses to TKIs than patients without this polymorphism [9]. This finding was confirmed by several studies [9–11]. However, Lee et al. suggested that *BIM* deletion polymorphism was not predictive of PFS for EGFR-TKIs [12]. The aim of this meta-analysis was to summarize all the available evidence and determine the predictive role of *BIM* deletion polymorphism for EGFR-TKIs in NSCLC.

## RESULTS

### Literature search

**Figure 1:**
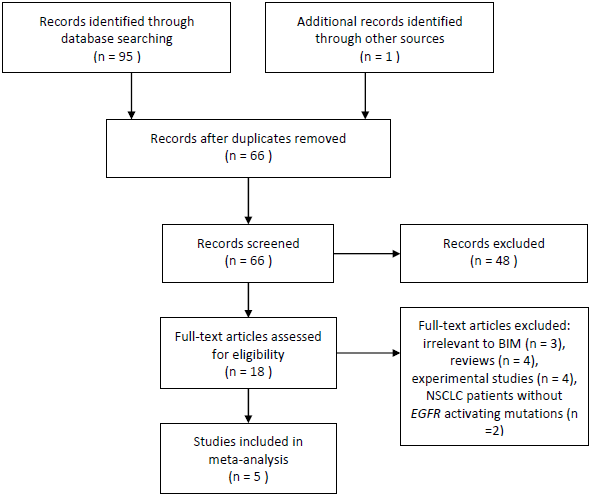
Flow of study identification, inclusion, and exclusion

The process of identifying studies is shown in Figure 1. A total of 95 publications were identified in the initial search, and 1 publication was identified from other source. Based on screening of titles or abstracts, 78 records were excluded. Full text articles were retrieved only for 18 publications and assessed for eligibility. Of these 18 publications, 13 publications were excluded. Finally, 5 studies were included in this meta-analysis.

### Study characteristics

Five retrospective cohort studies were included. All of the studies were conducted in Asian population (*n* = 951). Four studies included advanced NSCLC patients, and one study included NSCLC from early stage to advanced stage. Five studies reported the status of *EGFR* mutations, while two studies did not provide this information. All studies were assessed by Newcastle–Ottawa Scale (NOS). The quality scores ranged from 8 to 9, suggesting that the methodological quality was high. The characteristics of each study are presented in Table 1.

**Table 1:** Characteristics of the included studies

First author	Year	Study design	Ethnicity	Included patients	No. of patients	BIM deletion (%)	Age (years)	Gender (male/female)	Smoking status (smoker/non-smoker)	Adenocarcinoma (%)	EGFR mutation (Exon 19 deletion/L858R/others)	Clinical stage	PFS (median)	OS (median)	Covariants	Quality score
Ng	2012	Retrospective	Asian	NSCLC harboring EGFR-activating mutations	141	18.4	Wild Type: 59.0 ± 10.4 BIM deletion: 59.7 ± 9.8	Wild Type: 40/75 BIM deletion: 7/19	Wild Type: 20/94 BIM deletion: 5/20	Wild Type: 90.4 BIM deletion: 92.3	Wild Type: 65/37/13 BIM deletion: 11/12/3	III/IV/Recurrent	Wild Type: 11.9 months BIM deletion: 6.6 months HR = 2.08, 95% CI 1.29–3.38	NA	Age, gender, histology, smoking history, type of EGFR mutation by exon and specific mutation, stage, first- or second-line TKI therapy, race, country, TKI (gefitinib or erlotinib) and ECOG status	9
Lee JK	2013	Retrospective	Asian	NSCLC harboring EGFR-activating mutations	197	10.9	62 (Range: 31–85)	73/124	52/142	97	115/72/10	III/IV/Recurrent	Wild Type: 11.3 months BIM deletion: 11.9 months	NA	NA	9
Isobe	2014	Retrospective	Asian	NSCLC harboring EGFR-activating mutations	70	18.6	Wild Type: 65.4 ± 14.1 BIM deletion: 63.8 ± 6.7	Wild Type: 15/42 BIM deletion: 4/9	Wild Type: 16/41 BIM deletion: 4/9	Wild Type: 87.7 BIM deletion: 100	Wild Type: 27/28/2 BIM deletion: 6/7/0	IV/Recurrent	Wild Type: 17.8 months BIM deletion: 7.57 months HR = 3.99, 95% CI 1.86–8.55	Wild Type: 45.4 months BIM deletion: 39.2 months	Age, sex, performance status, brain metastasis, bone metastasis, pulmonary metastasis, liver metastasis, lymph node metastasis, EGFR mutation, EGFR-TKI response, smoking history	9
Zhao	2014	Retrospective	Asian	Advanced NSCLC harboring EGFR-activating mutations	352	12.8	Wild Type: 59 (Range: 32–81) BIM deletion: 59 (Range: 39–75)	Wild Type: 153/154 BIM deletion: 20/25	Wild Type: 82/225 BIM deletion: 15/30	Wild Type: 78.2 BIM deletion: 73.3	Wild Type: 107/90/3 BIM deletion: 15/12/1	IIIb/IV	Wild Type: 11 months BIM deletion: 4.7 months HR = 2.09, 95% CI 1.15–3.82	NA	Age, sex, smoke, EGFR mutational status	9
Zhong	2014	Retrospective	Asian	NSCLC harboring EGFR-activating mutations	191	15.5	Wild Type: 58.3 ± 11.7 BIM deletion: 60.0 ± 11.0	Wild Type: 105/140 BIM deletion: 23/22	Wild Type: 82/163 BIM deletion: 20/25	Wild Type: 82.9 BIM deletion: 77.8	Wild Type: 90/60/11 BIM deletion: 10/17/3	I to IV	Wild Type: 8.47 months BIM deletion: 6.69 months	Wild Type: 21.9 months BIM deletion: 21.9 months	Age, gender, smoking history, tumor stage, differentiation	8

NSCLC, non-small cell lung cancer; EGFR, epidermal growth factor receptor; BIM, Bcl-2-like protein 11; PFS, progression-free survival; OS, overall survival; TKI, tyrosine kinase inhibitor; NA, not available.

### Quantitative data synthesis

**Figure 2:**
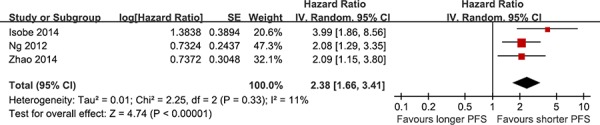
The prognostic role of *BIM* deletion polymorphism on PFS

All the studies reported the data of PFS. Except the study by Lee JK, all the studies suggested that *BIM* deletion polymorphism was associated with reduced PFS. Three studies reported adjusted hazard ratios (HRs) and 95% confidence intervals (Cis). Compared with *BIM* wild type, *BIM* deletion polymorphism was predictive of shorter PFS in NSCLC patients who were treated with EGFR-TKIs (adjusted HR = 2.38, 95% CI 1.66–3.41, *P* < 0.001; Figure 2). No significant heterogeneity was observed (*I* ^2^ = 11%).

## DISCUSSION

This systematic review and meta-analysis found that *BIM* deletion polymorphism was associated with a poor response to EGFR-TKIs in NSCLC patients. NSCLC patients with *BIM* deletion polymorphism exhibited shorter PFS when they received EGFR-TKIs.

As for overall survival (OS), two studies provided the median OS in *BIM* deletion polymorphism group and *BIM* wild type group. One studies indicated that patients with *BIM* deletion polymorphism had shorter OS than did those without BIM deletion polymorphism, while another study did not confirm this result. All the two studies did not provided statistical results, thus we did not do meta-analysis of OS.

The *BIM* deletion polymorphism contained a deletion of a 2903 bp fragment in intron 2. This deletion resulted in the preferential splicing of exon 3 over exon 4, which generated a *BIM* isoform that lacked the BH3 [8], thereby causing EGFR-TKIs resistant. They also reported that the addition of BH3-mimetic drugs could restore TKIs sensitivity [8]. Recently, Nakagawa et al. suggested the histone deacetylase (HDAC) inhibitor vorinostat could circumvent EGFR-TKI resistance in *EGFR*-mutant NSCLC cell lines and in xenograft models [13]. The combination of EGFR-TKIs plus a BH3-mimetic drug or vorinostat should be considered for the NSCLC patients with *BIM* deletion polymorphism in the future.

This meta-analysis had some advantages. First, this was the first meta-analysis which assessed the association between *BIM* deletion polymorphism and efficacy of EGFR-TKIs. Second, no significant heterogeneity was found in this meta-analysis. Third, the quality of the included studies was high. However, the limitations should also be acknowledged. First, there were only five studies included in this meta-analysis. Although all the studies reported PFS, only two studies provided OS. Thus, it was still unclear whether *BIM* deletion polymorphism was a prognostic marker of OS. Second, all of the studies were retrospective design, which were prone to bias (e.g., recall and selection bias). Therefore, prospective studies should be designed to validate the results of this meta-analysis. Third, we could not perform subgroup analyses by age, gender, smoking status, and *EGFR* mutations due to the insufficient data.

In conclusion, the *BIM* deletion polymorphism was significantly associated with poor response in NSCLC patients who received EGFR-TKIs treatment. The *BIM* deletion polymorphism could be a prognostic biomarker of EGFR-TKIs resistance in NSCLC.

## MATERIALS AND METHODS

### Literature search

PubMed, Embase, and the Cochrane Register of Controlled Trials were searched for relevant studies published up to 20 Apr. 2015. The following terms were used: (“NSCLC” or “lung cancer” or “non-small cell lung cancer”) and (“Bcl-2-like protein 11” or “Bcl-2-like 11” or BIM or BCL2L11”. No language restrictions were imposed. References from relevant articles, including review papers, were also reviewed.

### Study selection

Studies were included in the meta-analysis if they fulfilled the following inclusion criteria: 1) study design: cohort studies; 2) population: NSCLC patients with *EGFR* activating mutations; 3) intervention: EGFR-TKIs; 4) primary outcome: the effect of *BIM* deletion polymorphism on PFS. Abstract, case reports, review articles, experimental studies and commentary articles were excluded.

### Data collection and methodological quality assessment

The following data were extracted from each study: the first author, publication year, study design, ethnicity, included patients, number of patients, the percent of *BIM* deletion, sex, age, smoking status, the percent of adenocarcinoma, *EGFR* mutations, clinical stage of patients, reported PFS, and covariates controlled for multivariable analysis. Two reviewers independently extracted the relevant data. Any disagreement was resolved by consensus in meetings with all investigators.

Two reviewers completed the quality assessment independently. The NOS was used to evaluate the methodological quality, which scored studies by the selection of the study groups, the comparability of the groups, and the ascertainment of the outcome of interest [14]. We considered a study awarded 0–3, 4–6, or 7–9 as a low-, moderate-, or high-quality study, respectively. Discrepancies were resolved by consensus and discussion.

### Statistical analysis

We estimated the HR with 95% CI for primary outcome. The multivariable-adjusted HRs with 95% CIs were pooled in our analysis. We used a random effects model. Statistical heterogeneity among studies was evaluated using the Q and *I^2^* statistics. Publication bias was investigated by funnel plot if more than 10 studies were included. All statistical analyses were performed with Revman 5.1software (Nordic Cochrane Center, Copenhagen, Denmark).
